# The application of cognitive neuroscience to judicial models: recent progress and trends

**DOI:** 10.3389/fnins.2023.1257004

**Published:** 2023-09-22

**Authors:** Ni Zhang, Zixuan Zhang

**Affiliations:** ^1^Department of Sichuan University, Chengdu, China; ^2^School of Law, Sichuan University, Chengdu, China

**Keywords:** cognitive law, judicial bias, interpretability, legal prediction, cognitive computing

## Abstract

Legal prediction presents one of the most significant challenges when applying artificial intelligence (AI) to the legal field. The legal system is a complex adaptive system characterized by the ambiguity of legal language and the diversity of value functions. The imprecision and procedural knowledge inherent in law makes judicial issues difficult to be expressed in a computer symbol system. Current semantic processing and machine learning technologies cannot fully capture the complex nature of legal relations, thereby raising doubts about the accuracy of legal predictions and reliability of judicial models. Cognitive computing, designed to emulate human brain functions and aid in enhancing decision-making processes, offers a better understanding of legal data and the processes of legal reasoning. This paper discusses the advancements made in cognitive methods applied to legal concept learning, semantic extraction, judicial data processing, legal reasoning, understanding of judicial bias, and the interpretability of judicial models. The integration of cognitive neuroscience with law has facilitated several constructive attempts, indicating that the evolution of cognitive law could be the next frontier in the intersection of AI and legal practice.

## 1. Introduction

Cognitive neuroscience has developed rapidly and made significant advances since its emergence in the 1980s. Its application to legal fields in the 1990s led to the intersection of law and cognitive neuroscience. This has been termed ‘neurolaw’ (Hirsch, 2003). Cognitive solutions are designed to construct legal predictive models that are closely aligned with judicial reasoning, and to provide logical arguments to clarify and justify decision outcomes. The integration of cognitive science with psychology, AI, neuroscience, linguistics, anthropology and even natural philosophy can process large amounts of data and have a good understanding of the processes (Kelly, 2016).

The legal system fundamentally relies on the accumulation and interpretation of legal knowledge, which serves as the cornerstone for administering justice. Building on this foundation, judicial models have experienced significant advancements, especially in specialized areas. These areas include but are not limited to, legal translation, automated generation of legal documents, and the implementation of online dispute resolution systems. Hence, the intricate relationship between traditional legal knowledge and technological advancements is shaping a more robust and efficient judicial framework. In 2018, the China Supreme People’s Court initiated the ‘Intelligent Push System for Class Cases,’ and other courts have developed similar smart platforms. Despite these advances, current judicial models perform optimally in cases with relatively simple narratives and clear legal relationships, such as in tax and traffic areas. For more complex cases featuring complicated legal relations, the predictive accuracy of these models is not enough high to meet judicial requirements (Zuo, 2018). Furthermore, some biases are amplified through data-focused analyses within judicial models.

Extending the discussion, the ambiguity of legal language, the varying interpretations from different individuals, and the necessity of sensibility and common sense in judicial judgment all pose challenges. Tacit knowledge, procedural knowledge, and fuzzy knowledge are difficult to express via computer symbol systems. Cognitive computing, the interdisciplinary scientific investigation of the mind and intelligence, models human brain functions and mimics primary natural intelligence behaviors, which encompasses the ideas and methods of psychology, linguistics, philosophy, computer science, AI, neuroscience and anthropology (Thagard, 2009). This integration assists policymakers in deriving substantial insights from extensive amounts of unstructured data (Ludwig, 2013). As such, cognitive intelligence (CI) is anticipated to reduce discretionary bias and enhance the accuracy and explainability of judicial models. Based on the issues outlined above, this paper analyzes the challenges of the current AI-based judicial model in the first. Constructing such a model involves numerous complexities, which stem from the ambiguity of legal expression in judicial practices and the difficulty of integrating value judgments into the model. We will discuss the advancements in cognitive science as applied to various fields, including legal concept learning, semantic extraction, judicial data fusion, legal reasoning, judicial bias, and the interpretability of judicial models. The paper then reviews progress in cognitive methods relevant to these areas. Finally, it suggests that cognitive law will be the next interdisciplinary frontier, leveraging the advantages of cognitive computing to bridge the gap between AI and law.

## 2. Challenges of current judicial model

Despite significant advancements in computational power and perception ability, AI systems often lack common sense, logical reasoning, thinking, and adaptability, relying on partial and isolated data (Xinzhiyuan, 2018). Current semantic processing and machine learning technologies struggle to illuminate the complexity of legal relations, leading to suboptimal predictive performance. Furthermore, the current judicial model exhibits poor accuracy in judicial predictions due to incomplete legal data and imperfect techniques. It also has reduced reliability due to biased decisions that have not been effectively assessed by legal experts. The subsequent points outline the evident challenges present in the existing judicial model.

Firstly, the challenge of constructing a judicial model lies in managing substantial volumes of multi-source heterogeneous legal, integrating hundreds of data sources, and understanding their diverse formats (Gao, 2020). Judicial information encompasses a large volume of multi-source, heterogeneous, semi-structured, and unstructured data, originating from various resources such as judicial websites, court and procuratorate bulletins, annual judicial reports, press conferences, and various news media outlets. This information also includes statutes, regulations, and other legal documents, as well as previous cases. The diversity of sources, variable validity, irregular structure, and extraction difficulty of this information present notable challenges. Additionally, information represented in text, image, and video forms may suffer data loss during conversion. So, the question is: how do we effectively integrate these data?

Secondly, the performance of integrating various case features using machine learning to assist humans in finding similar cases is not as well as expected in the application of AI within the legal field (Zuo, 2018). In many models, cases are dissected into several factors including the case name, cause, involved parties, original statement, defendant’s argument, trial process, focal points of dispute, court’s investigation, court’s opinion, legal basis, judgment documents, and other fundamental information. The most prevalent method of constructing judicial models in China involves employing a large number of legal professionals to initially identify and label judicial cases, followed by the use of keywords to compare with system tags, ultimately resulting in the suggestion of similar cases (Li, 2018). However, cases identified through keyword-based and labeled similarities frequently fall short of expectations, requiring judges to invest considerable time and effort in reading and recognizing similar cases (Liu, 2022).

Thirdly, the judicial model will give some predictive suggestions, but the decision-making process is not transparent and cannot be exactly explained by legal person (Ding, 2020). As judicial argumentation fundamentally revolves around legal facts and values, the question arises: how do we integrate the values of traditional legal research into the judicial discretion model?

Finally, judges’ personal biases can affect the analysis due to the hidden biases in legal data. As a result, these biases may be reinforced by the judicial model, as the endogenous legal data also includes personal biases. Besides, legal data often includes private information, such as gender, age, race, drug use, socioeconomic status, support networks, education, images, addresses, and telephone numbers. Thus, how can we strike a balance between the openness and privacy of legal data? While it is often stated that the most effective machine learning models emulate human cognitive abilities, how can we minimize and prevent bias within judicial models?

## 3. Progress of cognitive methods applied to judicial model

The cognitive method is a computational model that relies on psychological concepts, illustrating how people approach problem-solving and task performance. This method involves simulating human problem-solving and mental processes within a computerized model (Burns, 2023). According to the American realism, the process of judicial decision-making is also influenced by the impulse and intuitive (Capurso, 1998), the prediction accuracy will be highly improved if the judging process can be understood with the help of cognitive algorithms.

### 3.1. Legal concept learning

Concepts do not have a bounded and perceivable referent; rather, they encapsulate linguistic and social experiences through various representational viewpoints (Borghi et al., 2017). In the context of the legal system, a discipline rich in diverse legal concepts, these ideas serve as foundational elements in the construction of judicial models. Further expanding on this notion, legal concepts can be best understood as mental representations that are crucial not only for legal reasoning and decision-making but also for understanding the ontology and epistemology of law itself (Jakubiec, 2022). However, these concepts are often articulated using abstract language. In the construction of a legal model, the cognitive aspects of legal concepts are thoroughly examined and analyzed. This is based on principles derived from both philosophy and cognitive psychology. The analysis encompasses the modeling of cognitive operators that form concepts, defining what constitutes cognitive concepts, and establishing structures for these cognitive concepts. In the broader scope of decision-making studies, granular computing emerges as a valuable paradigm for addressing higher types of uncertainty (Qin et al., 2023). When integrated with established cognitive concept structures, granular computing has been demonstrated to significantly improve the efficiency of concept learning (Li et al., 2015).

With cognitive methods rooted in theories from cognitive linguistics, particularly Categories and Prototypes, Schema Theory, and Legitimation Code Theory (LCT) (Bertoldi et al., 2014), a profound understanding of legal concepts can be achieved. Unlike computational thinking, which focuses on task decomposition, human beings can comprehend and explain concepts in combination with personal characteristics. A cognitive computing model is proposed to bridge the intension and extension of uncertain concepts, integrating human cognition of “from coarser to finer” and the computer’s information processing of “from finer to coarser” (Xu and Wang, 2019). The validity and efficiency of this bidirectional cognitive computing model are fundamentally aligned with human cognition.

Individuals discern the actual meaning of a specific concept when it is contextualized within the broader legal system. Consequently, legal concepts can be understood in the specific scenarios of this system. Cognitive science adopts a top-down method, breaking down complex cognitive processes into computational components, while most computational neural networks utilize bottom-up methods to illustrate the dynamic interactions between biological neurons. Edwards proposed concepts with cognitive structures can highlight some virtues called Higher-Level Unity approaches to concepts (Edwards, 2022). The Bayesian nonparametric model elucidates how a single experience can evolve into a concept (Allison, 2023). A concrete concept with a connection between conceptual aspect perception and the Radical Enactivism notion of attentional anchors, is proposed, which has in the context of science, technology, engineering, and mathematics etc (Rasmus, 2022). Cognitive concept maps, which is any visual representation of a person’s mental model for a concept, connecting knowledge by graphical tools, performs well while tackling sustainability issues (Watson et al., 2023). Legal concepts is mental representations with the analogical debating, when sentences are an emanation of the cognitive process of mapping, we will think about law as if it were a concrete, fragile entity (Jakubiec, 2022). Ashley (2017) used ontology-based system to represent the concepts and their relations, cognitive computing plays key roles in conceptual legal information retrieval.

### 3.2. Legal semantic extraction

AI can assist humans in dissecting and labeling facts, extracting legal features of judicial precedents acquiring feature weights, and enhancing system performance through feedback learning (Ashley, 2017). Legislative articles can be interpreted differently by different individuals, and precise guidance cannot always be gleaned from legal documents. Additionally, judicial language often holds ambiguity in judgments, rendering semantic extraction partially reliant on keynote searching in current models. While language acts as a medium for human communication, the primary focus of semantic extraction is to discern the underlying meaning rather than the surface-level language itself. Judicial language frequently contains open-ended and ambiguous meanings. Therefore, applying cognitive methods to semantic extraction can enhance our understanding of the implicit meanings behind such language, especially when these methods are integrated with the contextual background.

Furthermore, CI encompasses semantic understanding, knowledge representation, associative reasoning, and intelligent question answering, among other aspects. Humans possess multi-modal sequential memory and predictive abilities based on the perception of objects, time, and space. It is challenging for AI to emulate this human capability, although machine reading comprehension has outperformed humans in terms of precise matching indicators (Xinzhiyuan, 2018).

Frame semantics, which provides a schematic structure to describe the roles of participants and props in an event or state, is utilized for characterizing legal issues. Utilizing cognitive linguistic representation can thus improve the accuracy of legal information retrieval (Bertoldi et al., 2014). Building on this, a hybrid approach that combines blockchain and semantic web technologies has been proposed to validate learning outcomes in compliance with legal constraints (Nguyen et al., 2022).

A cognitive computing framework has been proposed to address challenges such as semantic understanding, knowledge acquisition, and judicial reasoning, particularly within the Chinese legal domain (Li et al., 2019). In a parallel development, ChatGPT has demonstrated the potential of machine learning in the legal field by successfully passing the Uniform Bar Examination in the top 10th percentile through transfer learning (Kimmel, 2023). These advancements indicate the growing role of AI and cognitive computing in reshaping the judicial landscape (Hong et al., 2020). Moreover, the integration of the legal feature vector and BERT has been utilized for matching similar legal cases, offering a more nuanced approach to case law analysis (Koniaris et al., 2023). Building on this, the application of Graph Convolutional Networks in judicial documents has further extended the capabilities of machine learning in the legal field, particularly by extracting criminal actions that are linked through two temporal relationships (Feng et al., 2022). Going even further, deep cognitive semantic research has been introduced, relying on knowledge graphs. Unlike traditional keyword-only searches, this approach not only considers the keywords but also their meaning within the search context. The system operates based on interactive iterative processes and incorporates the chaotic set of discovered facts for a more comprehensive analysis (Maksimov and Golitsyna, 2022). These progressive developments exemplify the ongoing advancements in applying machine learning techniques to various facets of the legal domain.

### 3.3. Judicial data fusion

Judicial information encompasses a vast array of multi-source heterogeneous semi-structured and unstructured data. When structuring this information, the facts and processes of judicial cases should be described accurately, ensuring complexity and completeness. Often, legal data is partial and isolated and cannot be directly applied in legal reasoning. New technologies based on cognitive science present a new approach to aggregating and understanding big data, with IBM’s Watson serving as a notable example (Chen et al., 2016). Watson’s legal module ROSS, called as “the world’s first artificially intelligent attorney,” which can understanding, retrieval and ranking legal information by integrating the multi-modal data like text, image and semantic, might align well with the meta-analytical system by considering broader social and jurisprudential contexts (Taal et al., 2016). A multi-layer semantic approach for digital forensics are proposed to automatic disposing the heterogeneous and unstructured data (Arshad et al., 2022).

Furthermore, some judicial cases may include outdated codes or errors, necessitating normalization and cleansing to transform the information into a formatted dataset suitable for analysis (Ma et al., 2016). To ensure the reliability and comprehensiveness of judicial information flow and to prevent information tampering, digital encryption and blockchain technology should be used throughout the process of judicial information storage, transmission, and cleaning.

Lastly, most factors in judicial models are extracted from written judgments. However, these judgments, which often do not disclose the argument process, constitute merely one data source for the judicial model and seldom provide logical arguments to clarify and justify the decision, and complex analysis of the background related to the case is often disregarded. Consequently, even experienced judges may struggle to obtain decision-making clues from trial judgments, making it an even more difficult task for machines to learn. The reasoning aspect should be included in the verdict, and judges’ key points should be summarized. It is recommended that additional case information such as indictments, trial processes, legal debates, and evidence determinations should be considered when constructing a judicial model.

### 3.4. Legal reasoning

Legal formalism posits that judges apply legal reasons to the facts of a case in a rational, mechanical, and deliberative manner (Capurso, 1998). However, a judge’s decision is often a complex argumentative process intertwined with rapid, intuitive judgments based on heuristics, and careful rational verification derived from legal provisions and judicial precedents Judicial intuition, which is developed through the accumulation of long-term legal knowledge, serves as the foundation for preliminary case identification (Guo and Wang, 2018). This initial identification is subsequently enhanced by a variety of technical methodologies and evidential reasoning to arrive at a final judgment. Complementing this, legal reasoning represents a unique blend of subjectivity and neutrality. To reconcile these opposing elements, a neutrosophic environment has been adopted in the realm of legal causal reasoning (JosRodolfo et al., 2021). Further deepening our understanding, legal reasoning encompasses various cognitive activities, including moral evaluation, problem-solving, and decision-making. In essence, legal reasoning can be viewed as a form of cognitive activation (Federico, 2020). These multiple layers of judicial intuition, technical analysis, and cognitive involvement together form the complex tapestry of legal decision-making.

Neuroscience, using brain imaging techniques, seeks to illustrate the role of a judge’s emotional and rational processes in decision-making (Johnson et al., 2016). Cognitive intelligence, which is higher stage of AI, can facilitate our understanding of the processes underlying judicial decisions by integrating legal rules, intermediate legal concepts and underlying values, is well-suited for complex analysis and can assist in revealing the principles of a judge’s trial (Ashley, 2017). Ashley (2017) proposed a judicial model by integrating values into the measures of case relevance and models of legal analogy. Cognitive methods are used to analyze lawyers’ moral decision-making by investigated the influence of the decision context and interceptive manipulation on the moral decision-making process (Angioletti et al., 2022).

Furthermore, legal reasoning involves analyzing statutes and cases to extract legal factors and relationships, and determining the likely verdict for a pending case. Judgments include discretionary factors such as the offender’s characteristics, subjective malignancy, objective consequences, and infringement methods, among others. However, additional discretionary elements like psychological, political, and social factors are often overlooked. The inherent legal logic is not revealed in current retrieval systems due to the absence of thorough legal arguments (Baker, 2018). An argument-based cognitive judicial model has been proposed, which employs a hybrid approach of human-machine collaboration. This model is designed to assess the relevance of judicial texts through interactive dialogue. In addition, it elucidates how various AI and Law techniques are executed within such systems (Ashley, 2017). This innovative model serves as a comprehensive platform that integrates human expertise with machine learning capabilities to enhance the efficacy and explainability of legal decision-making processes.

### 3.5. Judicial bias

Judicial decision-making is based on legislative statutes and precedent cases, and it requires judges to have the personal skills to manage relevant knowledge and data effectively. The personal values, attitudes, preferences, emotional responses, professional competence, ethical qualities, and group decision-making tendencies of judges, along with other external circumstances, play a decisive role in their court reasoning and judgment (Pound, 1984). Under certain circumstances, they may unconsciously exhibit bias or prejudice, particularly in intuitive decisions susceptible to heuristics and cognitive bias. Biases, such as racial and gender discrimination, may inevitably exist in judicial information (Alelyani 2021). Therefore, judicial models may also exhibit bias if they are trained on data that contain such biases. If the data fed into the system contain discriminatory information, this could exacerbate discrimination and inequality.

Moreover, when establishing a judicial model, the system developer’s choices in data selection, data analysis, and data presentation determine the system’s operation. While technology may appear value-neutral, the built-in value priority as a by-product of technology application can inadvertently reinforce certain social values. For instance, predictive policing software like COMPAS has been scrutinized for bias against Black individuals, who are significantly more likely to be incorrectly judged as “guilty” than their White counterparts. Though recent literature surrounding COMPAS has suggested that there is no bias and this is only a product of the data itself (Zhou et al., 2023), it is crucial to identify, highlight, and address inappropriate trends such as discrimination and incorrect value selection when constructing judicial models. Some measures used to mitigate bias include blind testing, blind verification, independent assessment, linear sequential unmasking (LSU), and the filler control method (Cooper and Meterko 2019; Meintjes et al., 2019).

Cognitive systems attempt to emulate aspects of human thinking, while adding the capacity to process large volumes of information and evaluate it without bias. Detecting and assessing bias is a crucial step toward creating more explainable models. Deliberative thought processing can correct initial errors in intuitive judgments. Encouraging judges to write more frequent opinions, reallocating decision-making authority, and implementing peer review and feedback can also reduce personal bias in judicial model construction (Gravett, 2017).

Judicial information may contain private data. Confidential words in a file or a website can be identified and transformed into meaningless words. For instance, part-of-speech (POS) tagging with neural networks is used to predict confidential words in judicial precedents and improve accuracy over previous models (Kanazawa et al., 2020). Additionally, several techniques, for example TIVA, Bayesian, Z-curve and so on (Drucker et al., 2016), have been proposed to detect and assess bias in machine learning models, especially in relation to humans’ cognitive bias. It is essential to consider the bias in some forensic science disciplines such as fingerprint examination, trace evidence, bullet comparison, and DNA analysis (Meintjes et al., 2019).

A judicial decision is a confluence of many values, and making the ranking of these values is a difficult task. Different judges may arrive at different decisions for the same case, and their judgment may be effected by many factors, such as societal impact, recognition from superiors or the public, promotion and salary adjustment, and timely case closure (Li, 2021). The value in the judicial discretion model may not solely represent an individual, but rather a synthesis of multiple individuals’ choices influenced by strategic considerations. When establishing a judicial model, the values-choosing and the value-ranks of legal experts should be presupposed according to moral orders to protect the majority’s rights. During this process, it is crucial to prevent the values of technical personnel or data providers from being forcibly imposed.

### 3.6. Explainable judicial model

Explainable artificial intelligence (AI) has become the new frontier in legal informatics. This is due to the fact that AI algorithms often lack transparency, a characteristic that should be viewed as an intrinsic property of an AI system rather than an external auditing process (Waltl and Vogl, 2018). The ‘black box’ nature of intelligent technology, wherein the developer’s personal values are obscured by technological packaging, poses a significant challenge to the construction of judicial models. The algorithm, refined through continuous adjustments and optimizations of training datasets, results in concealed and uncertain operational outcomes, making it challenging to discern the value choices and biases of technical staff. Even with identical data inputs, the results can differ greatly due to inconsistencies in the programming logic and learning models of the algorithm. Cognitive research investigates and emulates judges’ cognitive processes, and takes the ‘interpretation rules’ in the judge’s decisions as the foundational elements for constructing interpretation models.

Firstly, interpretability necessitates a system that legal experts, such as judge and lawyer, can in-depth analyze the decision-making processes. Making complex domain knowledge accessible and applicable for non-domain experts can enhance both the design and evaluation of such systems (Schoonderwoerd et al., 2022). Only when the processes of judicial argument, legal behavior motivations, and conclusions can be explained in a manner understandable to legal experts unfamiliar with AI techniques, can the judicial model gain practical acceptance.

Secondly, interpretability requires openness and transparency in constructing judicial models. The IEEE advocates for the disclosure of AI program source codes, and the provision of source code interpretations and other measures to promote resource openness and code transparency, thereby reducing the incidence of information disclosure errors within the program. The back-box can be glass-box by the way of providing explainable artificial intelligence services and using open-source tools, and strengthening supervision and addressing the responsibility to whom may access the legal data (Chinu, 2023). In addition, providers should disclose relevant information to potential customers (Porto, 2021). Interpretable models are used to construct interpretable AI-based digital forensics (Solanke, 2022).

Finally, interpretability requires the unveiling of the legal reasoning process. When legal reasoning is contextualized within the judicial background, cognitive computing provides explanations for legal models. Prakken and Ratsma (2022) proposed a case-based argumentation model through a defeasible argumentation game that contrasts information in favor of and against a plaintiff, which fully showcases the system’s argumentation process in decision-making.

## 4. Prospects of cognitive law

CI has become an inevitable trend to promote the interdisciplinary research of AI, brain cognition and neuroscience is the inspiration for the development of a new generation of AI (Zheng, 2019). Brain-inspired system may also advance classic computers from dada processors to the next generation of knowledge processors mimicking the brain (Wang et al., 2018). Cognitive computing refers to a system that can learn at scale, reason with purpose, and interact naturally with humans (Kelly, 2016), which aims to develop a coherent, unified, and universal mechanism inspired by the capabilities of the human mind. It contains decision, discovery and engagement and is the development of computer systems modeled on the human brain with the characteristic of integrating past experiences into itself (Modha et al., 2011). When countless concepts and cognitive dimensions are interconnected within a specific structure, a vast and intricate three-dimensional network structure system is formed, mirroring the network structure of the human brain. Convolutional Neural Networks (CNNs), Recurrent Neural Networks (RNNs), Graphical Neural Networks (GNNs), and Attentional Neural Networks (ANNs) have been extensively used. Computers are rapidly advancing in abilities such as speech, vision, answering queries, and decision making. Undoubtedly, reliable AI integrating cognitive neuroscience and computing neural networks will be the direction in the coming decades (Wang et al., 2021).

The legal system is a complex, adaptive entity. From a systems theory perspective (Ruhl et al., 2017), the legal system can be considered a multifaceted system composed of judges, prosecutors, lawyers, and legislators. Guided by statutes, regulations, and rules, the system operates through legislation, judgment, and mediation, with judicial feedback mechanisms such as appeal, retrial, and legislative evaluation in play. As the system continually debugs, it tends toward stability. The application of AI within the legal realm has expedited the disclosure and feedback of legal information, aiding in the discovery of effective judicial strategies, prediction of judicial decisions, and warning of legal risks. Legal information is open-textured, with rule conflicts, semantic vagueness, and the necessity for commonsense knowledge, making it challenging to express all types of tacit knowledge, process knowledge, and fuzzy knowledge through a computer symbol system.

CI emphasizes improving the ability of intelligent systems to understand data, express knowledge, and reason logically (Zhang and Pu, 2021). Cognitive neuroscience applied to law has helped to bridge the lack of in-depth analysis in decision-making of legal experts, such as judges and layers (Goodenough and Tucker, 2010). Neurolaw began in the late 1990s, the early research focus more on lie detection and evidence certification by exploring and seeking the biological mechanisms of human neural activity. With the development of cognitive science, CI has been widely used in legal area. CI offers tools with diagnostic, predictive, and predictive capabilities that are able to observe, learn and offer Insights, suggestion and even automatic actions. The fusion of CI and law engenders interdisciplinary subject, which is inclined to be called as cognitive law. Cognitive law enhances the understanding of legal concepts and behaviors, and improves the interpretability of intelligent judgments by utilizing cognitive intelligence. The generated networks become more complex in the dialogue between the customer and the machine, positioning the cognitive law as the next step in computational law. Judicial models based on cognitive neuroscience aim to discover deep-rooted rules of adjudication and improve the accuracy of judicial predictions. The progresses of recent application of neuroscience to legal field show that the prediction and accuracy have been improved by cognitive judicial model, more cognitive and value factors are considered, more explanations are provided, and the process of decision-making are analyzed by argument-based legal reasoning. The future of cognitive law will be more productive with the development of cognitive science and the closer cooperation between lawyers and scientists.

The research of CI is on the initial stage while cognitive law is also on the initial step. Cognitive law is not simple determinism, as many factors shape a human being (Goodenough and Tucker, 2010). More attention should be paid to potential bias and interpretability issues in judicial models, and procedures designed to reduce discrimination and increase transparency will be established before implementing judicial models.

## Author contributions

NZ: Investigation. ZZ: Writing – review & editing.

## Funding

The author(s) declare financial support was received for the research, authorship, and/or publication of this article. This work was supported by the National Social Science Fund of China: Research on intelligent discretion model and risk prevention mechanism in the construction of China smart court (Grant No. 21BFX031) and National Natural Science Foundation of China (Grant No. 62171303).

## Conflict of interest

The authors declare that the research was conducted in the absence of any commercial or financial relationships that could be construed as a potential conflict of interest.

## Publisher’s note

All claims expressed in this article are solely those of the authors and do not necessarily represent those of their affiliated organizations, or those of the publisher, the editors and the reviewers. Any product that may be evaluated in this article, or claim that may be made by its manufacturer, is not guaranteed or endorsed by the publisher.
